# An Automatic Epilepsy Detection Method Based on Improved Inductive Transfer Learning

**DOI:** 10.1155/2020/5046315

**Published:** 2020-08-03

**Authors:** Yufeng Yao, Zhiming Cui

**Affiliations:** ^1^The Institute of Intelligent Information Processing and Application, Soochow University, Suzhou 215006, China; ^2^Department of Computer Science and Engineering, Changshu Institute of Technology, Changshu 215500, China; ^3^Suzhou University of Science and Technology, Suzhou 215009, China

## Abstract

Epilepsy is a chronic disease caused by sudden abnormal discharge of brain neurons, causing transient brain dysfunction. The seizures of epilepsy have the characteristics of being sudden and repetitive, which has seriously endangered patients' health, cognition, etc. In the current condition, EEG plays a vital role in the diagnosis, judgment, and qualitative location of epilepsy among the clinical diagnosis of various epileptic seizures and is an indispensable means of detection. The study of the EEG signals of patients with epilepsy can provide a strong basis and useful information for in-depth understanding of its pathogenesis. Although, intelligent classification technologies based on machine learning have been widely used to the classification of epilepsy EEG signals and show the effectiveness. In fact, it is difficult to ensure that there is always enough EEG data available for training the model in real life, which will affect the performance of the algorithms. In view of this, to reduce the impact of insufficient data on the detection performance of the algorithms, a novel discriminate least squares regression- (DLSR-) based inductive transfer learning method was introduced which is on the basis of DLSR and the inductive transfer learning. And, it is applied to promote the adaptability and accuracy of the epilepsy EEG signal recognition. The proposed method inherits the advantages of DLSR; it can be more suitable for classification scenarios by expanding the interval between different classes. Meanwhile, it can simultaneously use the data of the target domain and the knowledge of the source domain, which is helpful for getting better performance. The results show that the improved method has more advantages in EEG signal recognition comparing to several other representative methods.

## 1. Introduction

At present, epilepsy has become a common disease in neurology. Its pathogenesis has not yet been fully elucidated, and it is usually defined as a chronic neurological disease caused by sudden abnormal discharge of brain neurons. The epileptic seizures are sudden and repetitive. Its onset is accompanied by clinical manifestations such as loss of consciousness, fainting, and twitching of extremities. It also has cognitive and mental disorders that seriously endanger patients' health, cognition, etc. [1, 2]. According to statistics, more than one percent of the world's population suffer from the disease [3], and there are approximately 9 million people with epilepsy in China. Therefore, the depth research and prevention of epilepsy play an indispensable role to alleviate the suffering of patients, improve the quality of life, and promote healthy development. As an important method for studying epilepsy, EEG uses electrodes to record the electrical activity of nerve cells in the brain, which contains a large amount of physiological and pathological information, and is of great importance in the clinical examination, location, and therapy of epilepsy. Therefore, for people with a tendency to epilepsy, automatic detection of epilepsy can analyze and screen the EEG signals of people at high risk for epilepsy, so as to realize early detection, perform timely intervention, and reduce the impact of epilepsy on people and the incidence of epilepsy. In view of this, it is of great value to study the epilepsy automatic detection algorithm based on EEG signals and develop an efficient and accurate epilepsy automatic detection system.

In fact, the study of automatic epileptic detection based on EEG signals has attracted extensive attention from scholars and experts at home and abroad since the 1970s. To predict the onset or preonset of epilepsy in the process of seizure detection, machine learning and pattern classification algorithms are generally applied to classify the EEG signals after extracting the characteristics of the time domain, frequency domain, time frequency domain, or nonlinear domain of the EEG. With the development of computer technology and digital signal processing technology, more and more methods are widely used in the study of seizure detection methods and have achieved certain research results, such as the Bayesian classifier [4], artificial neural network [5–9], support vector machine (SVM) [10–13], and fuzzy reasoning [14, 15]. For example, Obeyli extracted the Lyapunov exponential features of EEG signals and used probabilistic neural networks to classify EEG signals, so as to achieve high classification results [9]. Chan et al. extracted the time-frequency features of five subbands in the wavelet transform domain of epilepsy EEG signals and then used support vector machines and cluster regression models to recognize the onset of seizures. Aarabi et al. [14] extracted the features such as sample entropy, dominant frequency, average amplitude, and amplitude variation coefficient of intracranial EEG da4ta of patients with epilepsy and used the established fuzzy inference rules to fuse the EEG feature information for seizure detection. Although many of the above intelligent classification methods have shown the effectiveness of epilepsy EEG signal classification, they still face a challenge, that is, it is very hard to get enough EEG data for epilepsy to train the model in real life. Therefore, it has important practical value to explore how to use the knowledge acquired from related fields to enhance the classification performance of EEG data in the current scenario [16].

To solve the above challenges, a novel inductive transfer learning method based on discriminant least squares regression (TDLSR) was proposed. Meanwhile, it was applied to specific medical application scenarios, namely, epilepsy EEG signal classification, so as to relieve the effect of severe data shortage on the performance of the algorithms. Transfer learning is an effective way to transfer knowledge from related fields and is helpful of obtaining more information in the absence of sufficient data or information. It focuses on how to use the useful information from similar but different source domains to improve the classification result of the classifier in the target domain. When studying epilepsy EEG signal classification, the inductive transfer learning method naturally becomes the first choice because of insufficient labeled epilepsy EEG samples in the target domain sometimes. What is more, since the discriminant LSR is still based on least squares regression (LSR) [17], which can explain the importance of each feature in the prediction model based on the original data space, we introduced the inductive transfer learning method based on DLSR to use for epilepsy EEG signal classification. In summary, the innovations of this work are summarized as follows:

*Point 1*. The improved method is on the basis of the inductive transfer learning, but it has some difference from the traditional inductive transfer learning. The latter directly transfers the samples or features used in the source domain to the target domain for transfer learning, while the former uses a knowledge lever mechanism that transfers some knowledge from the source domain to the target domain. Then, the security of the data in the source domain can be well protected, and the data in the target domain and the knowledge in the source domain can be used simultaneously, so that the classification effect is better.

*Point 2*. The improved method expands DLSR that can be well applied to classification scenes into a novel method with certain transfer learning ability, so that it can be used in more complex scenes.

*Point 3*. The improved method inherits the characteristics of DLSR, that is, it can be better applied to classification scenarios by expanding the interval between different categories. And, it can transfer knowledge from the source domain, thus ensuring the rationality of its training model.

Finally, to better illustrate the basic idea of this study, the structure of the paper is as follows:

*Part 1*. Introduction to the research background, status, and significance of the thesis.

*Part 2*. The related work, including the related technology of epilepsy EEG signal detection and the epilepsy EEG signal classification based on transfer learning, is summarized in advance so that the following sections become more readable.

*Part 3*. The notations of the inductive transfer learning algorithm based on DLSR was introduced in detail.

*Part 4*. The reliability and validity of TDLSR algorithm in detection of epilepsy EEG signals based on a series of experimental were verified.

## 2. Related Work

Automatic epilepsy detection is based on signal processing technology and pattern recognition. It analyzes EEG data to identify the location and duration of seizures. Usually, the EEG signals collected during seizures was called seizure EEG, and the EEG signals collected in the nonseizure are called nonseizure EEG. The problem of automatic detection of epilepsy EEG is to effectively judge the above two types of EEG signals and identify seizures. The related detection technologies are introduced as follows.

### 2.1. The Related Technology of Epilepsy EEG Signal Detection

**Figure 1:**
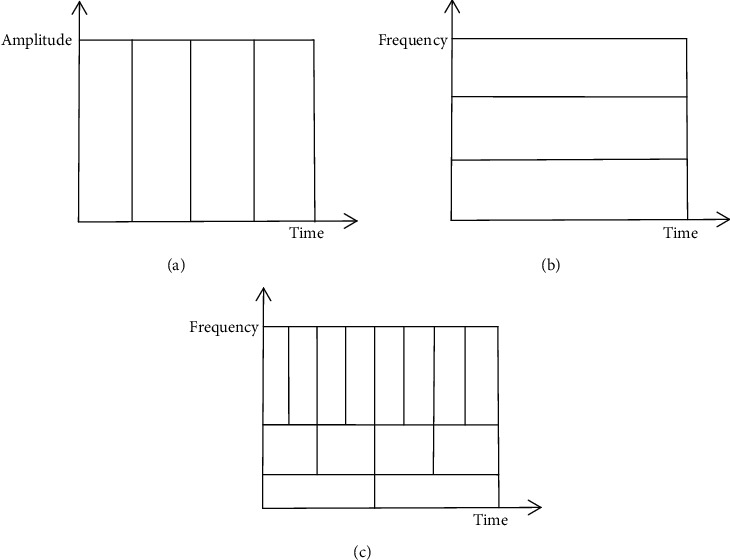
Comparison of time-domain analysis, frequency domain analysis, and time-frequency analysis. (a) Time-domain analysis. (b) Frequency analysis (Fourier transform). (c) Wavelet transform.

Because the EEG signal of epilepsy is easily interfered by many factors, it is very random, and it is a nonstationary signal, and its rule is generally difficult to grasp. Therefore, researchers often use quantitative analysis to extract characteristic information of epilepsy EEG signals. The existing methods of automatic epilepsy detection include the following:

1. Time-domain analysis: time-domain analysis is one of the earliest methods used in signal analysis. It analyzes the time-domain waveforms of EEG signals to study the difference between EEG waveform during seizures and EEG waveform during nonseizures, and directly extracts the waveform characteristics of the signals to distinguish the two types of EEG signals. The time-domain analysis method has the characteristics of intuitiveness and clear physical meaning, and it can reflect the important information in transient waveform such as spike wave and harp wave. The representative methods of time-domain analysis have a template matching method, time-domain waveform, or energy characteristics, etc. In 2001, Litt et al. [18] performed feature extraction on EEG signals based on time-domain analysis methods. In addition, researchers performed time-domain analysis of EEG signals to extract waveforms or energy features different from background activities and use amplitude, rhythm, period, and other parameters as classification criteria to identify epilepsy EEG signals. Gotman et al. performed a “half-wave” decomposition of EEG signals and then extracted EEG features, including average amplitude, duration, and coefficient of variation relative to the background, and set thresholds based on expert experience. The characteristic parameters are compared with the threshold to judge whether it is an epilepsy signal [19–21]. Time-domain analysis only performs statistical analysis from the time domain, and it is easy to miss other important changes in abnormal signals, such as slow waves
2. Frequency analysis: unlike time-domain analysis, which mainly analyzes the waveform characteristics of epilepsy EEG, frequency domain analysis analyzes the frequency characteristics of EEG. It recognizes different rhythms according to the frequency of brain waves. Each brain wave of different rhythms corresponds to epilepsy EEG signals in different time periods or different parts of the brain [22]. Frequency domain analysis is based on the Fourier transform and is mainly used for power spectrum analysis of EEG signals. It performs the Fourier transform on the EEG signal to obtain its frequency components and spectrum distribution and extracts the corresponding EEG features in the frequency domain for epilepsy detection and recognition. Representative methods include power spectrum estimation, autoregressive (AR) model spectrum estimation, and higher order spectrum [23]. Among them, the power spectrum estimation transforms the EEG signal whose amplitude changes with time into the EEG spectrum chart with power varying with frequency and analyzes the distribution and change of each frequency band of the EEG signal intuitively and quantitatively [24, 25]. Although frequency domain analysis can provide a lot of effective information, allowing researchers to detect epilepsy based on the frequency domain characteristics of EEG, the overall spectrum of the signal obtained by the Fourier transform neither can reflect the local characteristics of the signal nor can reflect the signal frequency component changes with time. Therefore, the detection results obtained by frequency domain analysis are not very satisfactory and are greatly restricted in practical applications
3. Time-frequency analysis: the epilepsy EEG is a typical nonstationary signal, which contains not only the waveform parameter characteristics in the time domain but also the energy distribution characteristics in the frequency domain. However, neither the above two methods can fully extract the transient characteristics and information of the EEG signals and can get the ideal results. With the development of digital signal theory and methods, the time-frequency analysis method combining time domain and frequency domain is widely used in the analysis of nonstationary EEG signals. It can obtain time and frequency domain information at the same time and capture transient information of EEG. In recent years, more and more studies have adopted time-frequency analysis methods to analyze EEG signals, among which various wavelet change methods are represented. The wavelet transform uses the translation and expansion of the window function to implement a wide time window for the low-frequency components of the signal and a narrow time window for the high-frequency components to complete the multiscale analysis of the signal. This analysis method conforms to the laws of nature and has a good ability to characterize the local characteristics of the signal [26]. It can capture the transient characteristics of the EEG signal and accurately locate it in the time and frequency domains. In addition to wavelet transform, commonly used time-frequency analysis methods also include empirical mode decomposition [27–30], the Wigner-Ville distribution [31, 32], and the Stockwell transform [33, 34]. However, most of these time-frequency analysis methods can only be used for multiresolution analysis of the original signal and then need to be combined with other algorithms to achieve the feature extraction and selection of EEG.  Figure 1 shows the comparison of time-domain analysis, frequency domain analysis, and wavelet transform analysis.
4. Observe the above figures, it is easy to draw a conclusion that the time-frequency analysis can provide more useful information compared to time-domain analysis and frequency domain analysis. In Figure 1(c), the wavelet transform improves the time resolution at the high frequency of the signal by changing the time window and improves the frequency resolution at the low frequency, which has a better classification effect
5. Nonlinear dynamic analysis: nowadays, with the progress of nonlinear dynamics theory, researchers are devoted to studying the nonlinear of EEG signals to solve the problem of automatic detection of epilepsy. Using nonlinear dynamics theory methods for EEG signal analysis, various nonlinear features of EEG signals can be extracted to distinguish epilepsy EEG signals from normal EEG signals. This provides some new research ideas for automatic epilepsy detection technology. Kannathal et al. used different entropies to measure the chaotic characteristics of EEG signals and used them as EEG features to distinguish EEG signals in different periods [35], including the Shannon entropy, Renyi entropy, Kolmogorov-Sinai entropy, and approximate entropy. The results show that the complexity of the EEG signal in patients with epilepsy during the intermittent period is higher than that during the seizure period, that is, the complexity of the EEG signal during the seizure is reduced, and the bet value is less than the normal EEG signal. Although nonlinear analysis can reflect the dynamic mechanism of seizures well, most of the nonlinear features are computationally intensive and generally time-consuming, which is not suitable for real-time epilepsy automatic detection systems.

### 2.2. The Epilepsy EEG Signal Classification Based on Transfer Learning

**Figure 2:**
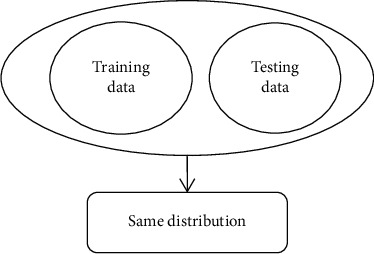
Scene suitable for the traditional method.

**Figure 3:**
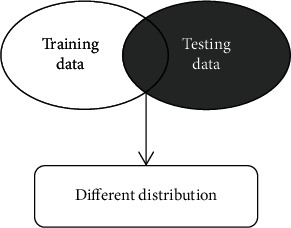
New challenge for the traditional methods.

**Figure 4:**
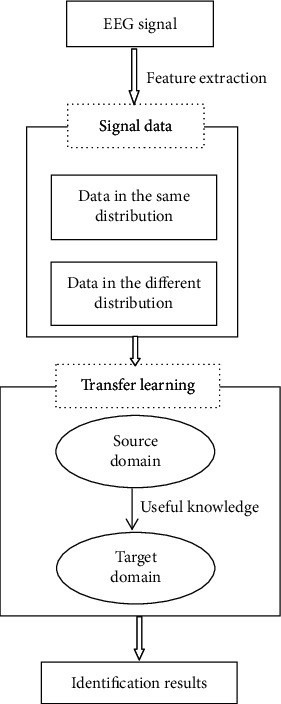
Framework of adaptive recognition for epileptic EEG signals based on transfer learning.

Traditional classification methods use a large amount of data with label information to train a decision function and then use this function to classify and identify test samples with unknown label information. However, these classification methods all have a presupposition: training data and test data need to obey the same distribution characteristics, as shown in Figure 2. For the differences in the distribution of training samples and test samples as described above, the performance of the traditional methods significantly decrease, as shown in Figure 3. In response to this challenge, transfer learning is a promising research direction. Transfer learning focuses on knowledge transfer problems that are similar to different domains or have different data distributions. It enhances the performance of the classifier used for target area recognition by learning useful knowledge from the source domain. According to whether the target domain used contains samples with labeled information, transfer learning techniques are divided into three categories: inductive transfer learning method, direct transductive transfer learning method, and unsupervised transfer learning method [36].. In the paper, we will focus on an inductive transfer learning method with good performance, that is, inductive transfer learning method based on discriminant least squares regression (TDLSR). And, its application and actual effect in EEG signal detection of epilepsy will be studied. The framework structure of epilepsy EEG signal detection based on transfer learning theory is given, as shown in Figure 4.

In short, transfer learning is to transfer the knowledge (useful knowledge) from the source domain with a large amount of labeled data for learning in the target domain with no or little labeled data, thereby improving the training quality of the target domain. This can reduce the workload of collecting labeled data in the target domain.

## 3. The Inductive Transfer Learning Algorithm Based on DLSR

To better describe the algorithm proposed in this paper, Table 1 gives a detailed description of the symbols in the algorithm.

Since DLSR is a nontransforming algorithm, the symbols in Table 1 refer to the parameter variables of the original training sample.

### 3.1. The Least Squares Regression

As a widely used method based on statistical theory, LSR has become a typical method. LSR uses the Frobenius norm to constrain the matrix of representation coefficients. In the paper, to expand the classification ability of the LSR algorithm, we preconstructed the binary label matrix *Y* corresponding to the training samples *X*, so that it can better cope with more complex classification scenarios. The *j*th column of *Y* indicates that only the data belonging to the *j*th class corresponds to an element equal to 1, and all other elements are 0. Then, the objective function of LSR can be redefined as follows:

(1) $$\begin{array}{c} \underset{Z}{\arg\min}{\left\|XZ-Y\right\|}_{\text{F}}^{2}+\lambda {\left\|Z\right\|}_{\text{F}}^{2}. \end{array}$$

Since the LSR has an analytical solution, which can be easily obtained, formula (1) can be rewritten as:

(2) $$\begin{array}{c} J\left(Z\right)=\underset{Z}{\arg\min}{\left\|XZ-Y\right\|}_{\text{F}}^{2}+\lambda {\left\|Z\right\|}_{\text{F}}^{2}. \end{array}$$

Then, the solution process of the analytical solution of LSR is as follows: let

(3) $$\begin{array}{c} \begin{array}{c} \frac{\partial J\left(Z\right)}{\partial Z}=0,\\ ⟹2X^{\text{T}}XZ-2X^{\text{T}}Y+2\lambda Z=0,\\ ⟹\left(X^{\text{T}}X+\lambda I_{d}\right)Z=X^{\text{T}}Y. \end{array} \end{array}$$

Finally, the analytical solution of LSR can be obtained as

(4) $$\begin{array}{c} Z={\left(X^{\text{T}}X+\lambda I_{d}\right)}^{-1}X^{\text{T}}Y. \end{array}$$

### 3.2. The Discriminate Least Squares Regression

As we know, LSR can be directly used for classification tasks. However, since the interval between any two different classes in the constructed binary class label matrix is $\sqrt{2}$, the DLSR proposed in literature [37] introduces the relaxation technique into the LSR so as to expand the interval between the two data from different classes. To improve the compactness of the classification task, DLSR will comprehensively consider the class factors and build an indicator matrix *B* on the basis of the binary label matrix *Y* of the sample. Each element of matrix *B* is defined as follows:

(5) B=ij+1,if yi=1,−1,otherwise.

In essence, each element of matrix *B* represents the offset direction of the corresponding class label. Then, *ε* relaxation on each element of *Y* is performed, and the amount of *ε* relaxation through the matrix *W* is recorded. Then, the objective function of DLSR can be expressed as

(6) $$\begin{array}{c} \underset{s.t.W\geq 0}{\min}{\left\|XZ+{ep}^{\text{T}}-\left(Y+B\Theta W\right)\right\|}_{\text{F}}^{2}+\lambda {\left\|Z\right\|}_{\text{F}}^{2}. \end{array}$$

The objective function of DLSR is a convex optimization problem. However, it cannot directly optimize the solution. Literature [37] adopts an alternative optimization strategy and ensures that a closed solution is obtained at each step. The specific derivation process is as follows:

(1) Fix *W*, and update *Z* and *p*. Let *L* = *Y* + *B*Θ*W*; formula (6) can be rewritten as
  (7) $$\begin{array}{c} J\left(Z,p\right)=\underset{Z,p}{\arg\;\min}{\left\|XZ+{ep}^{\text{T}}-L\right\|}_{\text{F}}^{2}+\lambda {\left\|Z\right\|}_{\text{F}}^{2}. \end{array}$$
According to the optimization theory, we take a partial derivative of *p*, namely
  (8) $$\begin{array}{c} \begin{array}{c} \frac{\partial J\left(Z,p\right)}{\partial p}=0,\\ ⟹Z^{\text{T}}X^{\text{T}}e+{pe}^{\text{T}}e-L^{\text{T}}e=0,\\ ⟹p=\frac{\left(L^{\text{T}}e-Z^{\text{T}}X^{\text{T}}e\right)}{N}. \end{array} \end{array}$$
Furthermore, we find the partial derivative of *Z*, and we can get
  (9) $$\begin{array}{c} \begin{array}{c} \frac{\partial J\left(Z\right)}{\partial Z}=0,\\ ⟹2X^{\text{T}}XZ-2X^{\text{T}}\left({ep}^{\text{T}}-L\right)+2\lambda Z=0,\\ ⟹X^{\text{T}}XZ-X^{\text{T}}\left(e\frac{\left(e^{\text{T}}L-e^{\text{T}}XZ\right)}{N}-L\right)+\lambda Z=0,\\ ⟹X^{\text{T}}\left(I_{N}-\frac{{ee}^{\text{T}}}{N}\right)XZ-X^{\text{T}}\left(I_{N}-\frac{{ee}^{\text{T}}}{N}\right)L+\lambda Z=0,\\ ⟹Z={\left(X^{\text{T}}HX+\lambda I_{d}\right)}^{-1}X^{\text{T}}HL \end{array} \end{array}$$
(2) Fix *Z* and *p*, and update *W*. Let *G* = *XZ* + *ep*^*T*^ − *Y*; then, *W* can be solved as follows:
  (10) $$\begin{array}{c} \underset{\begin{array}{c} W\\ s.t.W\geq 0 \end{array}}{\arg\;\min}{\left\|G-B\Theta W\right\|}_{\text{F}}^{2}. \end{array}$$

According to literature [37], the Frobenius norm square of a matrix can be solved element by element, so Eq. (10) can be equivalent to solve *N* × *C* subproblems. For element *W*_*ij*_ in row *i* and column *j*, there can be

(11) $$\begin{array}{c} \underset{s.t.W_{ij}\geq 0}{\underset{W_{ij}}{\min}{\left(G_{ij}-B_{ij}W_{ij}\right)}^{2}}, \end{array}$$

where *G*_*ij*_ and *B*_*ij*_ represent the *j*th element of the *i*th row of the matrix *G* and matrix *B*, respectively and satisfy *B*_*ij*_^2^ = 1. Then, we can get

(12) $$\begin{array}{c} {\left(G_{ij}-B_{ij}W_{ij}\right)}^{2}={\left(B_{ij}G_{ij}-W_{ij}\right)}^{2}. \end{array}$$

And, because each element of *W* satisfies *W*_*ij*_ ≥ 0, formula (12) can be written as

(13) $$\begin{array}{c} W_{ij}=\max\left(B_{ij}G_{ij},0\right). \end{array}$$

Therefore, the final solution formula of *W* is:

(14) $$\begin{array}{c} W=\max\left(B\Theta G,0\right). \end{array}$$

According to the above derivation, Algorithm 1 gives a detailed description of the DLSR algorithm.

### 3.3. The Inductive Transfer Learning Algorithm Based on DLSR

Most inductive transfer learning algorithms are implemented by directly learning from the data in the source domain through some classes. However, in the paper, we used a knowledge-based inductive transfer learning framework instead of raw data to study inductive transfer learning methods based on source domain knowledge. Inspired by this, an inductive transfer learning algorithm based on DLSR was introduced. Its objective function is

(15) $$\begin{array}{c} \underset{s.t.W\geq 0}{\underset{Z,p}{\min}{\left\|XZ+{ep}^{\text{T}}-\left(Y+B\Theta W\right)\right\|}_{\text{F}}^{2}}+\lambda {\left\|Z\right\|}_{\text{F}}^{2}+\eta {\left\|Z-Z_{s}\right\|}^{2}. \end{array}$$

It can be found from the formula (15) that the first two items directly inherit the DLSR for learning in the target domain. The third item is used to transfer the knowledge *Z*_*s*_ of the source domain to the target domain. When *η* = 0, DSLR is DSLR.

In short, TDLSR summarizes DLSR from the perspective of transfer learning, but it has more transfer learning capabilities than DLSR and has better applicability. Similar to DLSR, the objective function of TDLSR can also be solved using an alternate optimization strategy. The specific derivation process is as follows:

(1) Fix *W*, and update *Z* and *p*. Let *L* = *Y* + *B*Θ*W*; formula (15) can be rewritten as
  (16) $$\begin{array}{c} J\left(Z,p\right)=\underset{Z,p}{\arg\;\min}{\left\|XZ+{ep}^{\text{T}}-L\right\|}_{\text{F}}^{2}+\lambda {\left\|Z\right\|}_{\text{F}}^{2}+\eta {\left\|Z-Z_{S}\right\|}_{\text{F}}^{2}. \end{array}$$
According to the optimization theory, we take a partial derivative of *p*, namely
  (17) $$\begin{array}{c} \begin{array}{c} \frac{\partial J\left(Z,p\right)}{\partial p}=0,\\ ⟹Z^{\text{T}}X^{\text{T}}e+{pe}^{\text{T}}e-L^{\text{T}}e=0,\\ ⟹p=\frac{\left(L^{\text{T}}e-Z^{\text{T}}X^{\text{T}}e\right)}{N}. \end{array} \end{array}$$
Furthermore, we find the partial derivative of *Z*, we can get
  (18) $$\begin{array}{c} \begin{array}{c} \frac{\partial J\left(Z\right)}{\partial Z}=0,\\ ⟹2X^{\text{T}}XZ-2X^{\text{T}}\left({ep}^{\text{T}}-L\right)+2\lambda Z+2\eta \left(Z-Z_{S}\right)=0,\\ ⟹X^{\text{T}}XZ-X^{\text{T}}\left(e\frac{\left(e^{\text{T}}L-e^{\text{T}}XZ\right)}{N}-L\right)+\lambda Z+\eta \left(Z-Z_{S}\right)=0,\\ ⟹X^{\text{T}}\left(I_{N}-\frac{{ee}^{\text{T}}}{N}\right)XZ-X^{\text{T}}\left(I_{N}-\frac{{ee}^{\text{T}}}{N}\right)L+\left(\lambda +\eta \right)Z-\eta Z_{S}=0,\\ ⟹Z={\left(X^{\text{T}}HX+\left(\lambda +\eta \right)I_{d}\right)}^{-1}\left(X^{\text{T}}HL+\eta Z_{S}\right) \end{array} \end{array}$$
(2) Fix *Z* and *p*, and update *W*. Let *G* = *XZ* + *ep*^*T*^ − *Y*; then, *W* can be solved as:
  (19) $$\begin{array}{c} \underset{\begin{array}{c} W\\ s.t.W\geq 0 \end{array}}{\arg\;\min}{\left\|G-B\Theta W\right\|}_{\text{F}}^{2}. \end{array}$$

Similar to the way of solving *W* in DLSR, the final solution formula of *W* is:

(20) $$\begin{array}{c} W=\max\left(B\Theta G,0\right). \end{array}$$

According to the above derivation process, Algorithm 2 gives a detailed description of the TDLSR algorithm.

**Figure 5:**
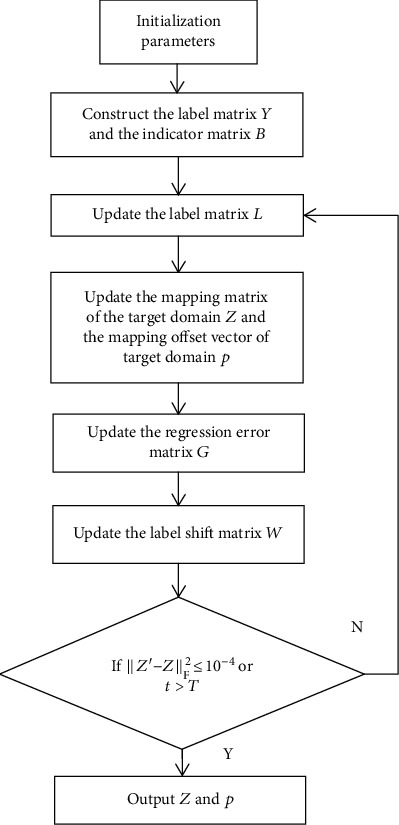
The process of the TDLSR algorithm.

To understand the TDLSR algorithm more clearly, Figure 5 shows the specific process of the TDLSR.

## 4. Experimental Research

### 4.1. The Environment and Parameter Settings

To evaluate the performance of the TDLSR, a lot of experiments were carried out based on EEG signal recognition. Experiments will be conducted from two aspects: (1) the comparison experiments with classic LSR, SRC, RLR, DLSR, and SVM and (2) the comparison experiments with related methods with transform ability, such as AuSVM, Tr-Adaboost, TSVM, and LMPROJ. The hardware environment used in the experiment is Intel (R) Core(TM) I7-9700 3.0 GHz×2, 8 G RAM, and the software environment is Windows 10 64 bit, MATLAB R2012b.  Table 2 shows the parameter settings of the above algorithms.

### 4.2. Experimental Dataset

In the experiments, the EEG dataset used for epilepsy is the Bonn dataset [38, 39], which was collected by Andrzejak et al. at an epilepsy center at the University of Bonn. The EEG dataset contains five datasets, denoted by A to E. This dataset compares the EEG of the patient during the onset and nononset period with the EEG of the normal person. Dataset A and dataset B are EEG signals collected by healthy testers with their eyes open and closed. Dataset C and dataset D are the EEG signals collected by epilepsy patients outside and inside the lesion during the seizure period, and dataset E is the EEG signals collected by the patients in dataset C and dataset D during the seizure. Each of the 5 datasets includes 100 single-channel EEGs (that is 100 samples), the sampling frequency is 173.61 Hz, each segment of the signal collects 4097 frequency points, and each EEG segment lasts 23.6 s.

In our experiments, the use of the Bonn dataset is significantly different from many previous works. We constructed 8 subdatasets from the original 5 datasets to simulate different scenarios in the experiments. The source and target domains of the 8 subdatasets of experiments are composed of the partial data extracted from the 5 sets. We randomly select 75% of the data from a certain dataset as the source domain, and the remaining 25% as the target domain. The sample data of source and target domains of subdatasets 1-4 are derived from the same distribution, but the samples taken are different. The sample data of the source domain and the target domain of subdatasets 5-8 have different distributions. Finally, for the data in the target domain, 20% is randomly selected for testing, and the remaining 80% is used for training. In addition, the training and test datasets do not contain the same samples and are independent of each other.  Table 3 lists the detailed information of the 8 subdatasets.

### 4.3. Experimental Results and Analysis

To verify the effectiveness of TDLSR in epilepsy EEG data recognition, the comparative experiments were conducted among the classic algorithms. The experimental results are shown in Tables 4 and 5, respectively.

As shown in the above experimental results, the conclusions are summarized as follows:

1. In the case of the same distribution of the source domain dataset and the target domain dataset, both the nontransfer learning algorithms and the transfer learning algorithms achieve good classification results. For the datasets with certain differences in distribution, the classification effects of algorithms without transforming abilities are quite different, and the experimental results of the algorithms in Table 5 are generally better than results of the algorithms in Table 4
2. As a whole, the performance of TDLSR introduced in this paper is obviously superior to all other algorithms. It means that by using the knowledge transferred from the source domain to the target domain, the TDLSR algorithm obtains better performance and becomes effective for epilepsy EEG recognition
3. Comparing the performance of the algorithms in Table 4, it can be found that DLSR has the best performance, while SRC and SVM have poor performance. This is because DLSR expands the ability to distinguish between classes by using the class information in the label space for the classification task. As the results shown in Table 5, the TDLSR algorithm performs best. This is because it not only inherits the advantages of DLSR by increasing the interval between different classes but also transfers more useful information from the source domain to the target domain. It has stronger transfer learning ability than several other algorithms. And because the TDLSR method requires fewer parameters to adjust, it is easier to use, and the stability and fault tolerance are stronger than the transfer learning algorithms such as LMPROJ.

**Figure 6:**
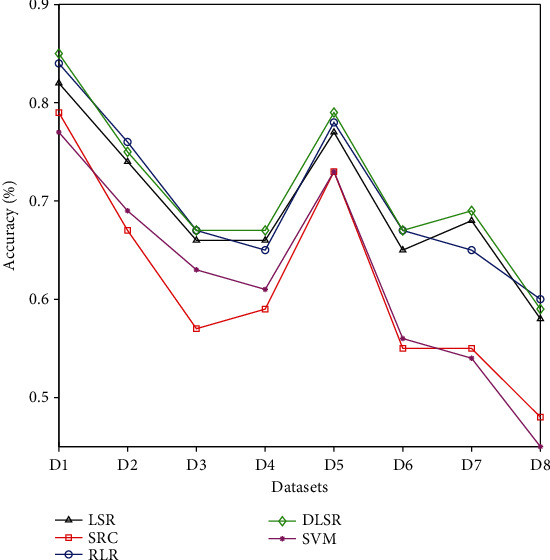
The average classification accuracy of all nontransfer learning algorithms.

**Figure 7:**
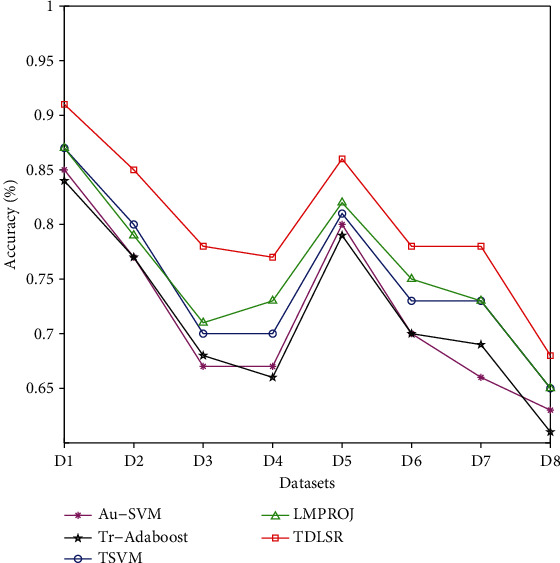
The average classification accuracy of all transfer learning algorithms.

In addition, to further observe and compare the overall classification performance of all algorithms, Figures 6 and 7 also give the average classification accuracy of each algorithm on all datasets.

Observing Figures 6 and 7, it can be seen that the classification accuracy of the algorithms on the same distributed dataset is higher than the result on the different distributed dataset. Secondly, compared with the traditional nontransfer learning algorithms, the algorithm with transfer learning ability has more advantages in classification performance, and the TDLSR has a significant improvement in classification performance. Finally, for all datasets with different classes, the performance of all algorithms decreases continuously as the number of categories increases. This is because as the number of classes increases, the information in the label space becomes more complicated, and learning the information in the label space becomes more difficult.

Meanwhile, to further verify the reliability and stability of the algorithms, we randomly added 15% white Gaussian noise to the data in the source domain to prove that the algorithm in this paper can adapt to more complex scenarios. The experimental results are shown in Tables 6 and 7, respectively.

Through the above experimental results, it can be found that the classification performance of all nontransfer learning algorithms under noisy conditions decreases more. The reason is that they cannot obtain useful knowledge from noisy data (source domain) for classification. However, all the algorithms in Table 7 can transfer some useful knowledge from the source domain for classification in the target domain, so their performance is better than the results in Table 6.

In summary, the TDLSR algorithm introduced in the paper is superior to the other algorithms in the detection of epilepsy EEG signals. And, it is easy to learn and train, has high stability, and shows certain advantages compared with other intelligent algorithms.

## 5. Conclusion

To solve the problem of serious shortage of training data in the current scene and improve the accuracy of classification, a novel DLSR-based inductive transfer learning algorithm (TDLSR) was introduced for the detection of epilepsy EEG signals. It can take advantage of both inductive transfer learning and DLSR. On the one hand, it can not only protect the security of the source domain data but also use the data of the target domain and the knowledge of the source domain to get better performance. On the other hand, it inherits the DLSR's characteristics of being more suitable for real classification scenarios by expanding the interval between different categories. Therefore, compared with DLSR, the new algorithm not only enhances the ability of transfer learning but also ensures that the model is more reasonable. The results reflect that the improved algorithm has more advantages in epilepsy EEG signal classification compared with the traditional algorithms. However, it is found that the results is easily affected by the parameters. In a word, the quality of the parameter selection will directly affect the final detection accuracy. Therefore, to obtain higher detection accuracy, it is worth to further study the characteristics of the EEG signal to guide the setting range of the parameters in the transfer learning algorithm.

## Figures and Tables

**Algorithm 1:**
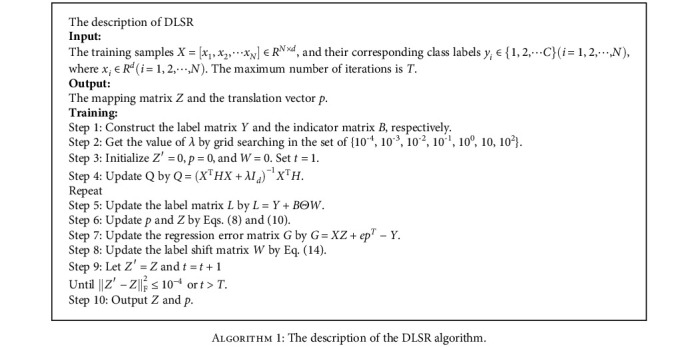
The description of the DLSR algorithm.

**Algorithm 2:**
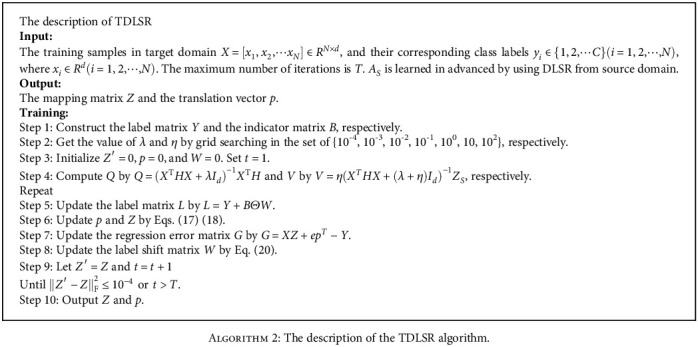
The description of the TDLSR algorithm.

**Table 1:** Symbol description.

Symbol	Description
*X*	Training matrix of the target domain, *X* = [*x*_1_, *x*_2_, ⋯, *x*_*N*_] and *x*_*i*_ ∈ *R*^*d*^
*N*	Total number of training samples of the target domain
*d*	Dimension of sample
*C*	The number of classes
*Y*	The binary label matrix corresponding to the training samples in the target domain. The *j*th column of *Y* indicates that only the data belonging to the *j*th class corresponds to an element equal to 1, and all other elements are 0
*B*	Indicator matrix constructed based on *Y*
*W*	Label offset matrix of the target domain
*Z*	Mapping matrix of target domain
*Z*_*S*_	Mapping matrix of source domain
*e*	Vector representing all 1, *e* ∈ *R*^*N*^
Θ	Hadamard product operator for matrix
*p*	The mapping offset vector of target domain
*λ*, *η*	The regularization parameter

**Table 2:** The parameter settings in the experiments.

Methods	Parameter settings
LSR	The regularization parameter *λ* ∈ {10^−4^, 10^−3^, 10^−2^, 10^−1^, 10^0^, 10, 10^2^}
SRC	The regularization parameter *α* ∈ {10^‐3^, 5 × 10^‐3^, 10^‐2^, 2 × 10^‐2^, 5 × 10^‐2^, 10^‐1^}
RLR	The regularization parameter *λ* ∈ {10^−4^, 10^−3^, 10^−2^, 10^−1^, 10^0^}; the heart parameter *δ* ∈ {2^‐5^, 2^‐4^, 2^‐3^, ⋯, 2^3^, 2^4^, 2^5^}
DLSR	The regularization parameter *λ* ∈ {10^−4^, 10^−3^, 10^−2^, 10^−1^, 10^0^, 10, 10^2^}
SVM	The penalty factor *c* ∈ {2^‐5^, 2^‐4^, 2^‐3^, ⋯, 2^3^, 2^4^, 2^5^}, the kernel parameter *δ* ∈ {2^‐5^, 2^‐4^, 2^‐3^, ⋯, 2^3^, 2^4^, 2^5^}
TSVM	The Lagrange multiplier upper bound *c* ∈ {2^‐5^, 2^‐4^, 2^‐3^, ⋯, 2^3^, 2^4^, 2^5^}, the kernel parameter *δ* ∈ {2^‐5^, 2^‐4^, 2^‐3^, ⋯, 2^3^, 2^4^, 2^5^}
Au-SVM	The penalty factor *c* ∈ {2^‐5^, 2^‐4^, 2^‐3^, ⋯, 2^3^, 2^4^, 2^5^}, the kernel parameter *δ* ∈ {2^‐5^, 2^‐4^, 2^‐3^, ⋯, 2^3^, 2^4^, 2^5^}
Tr-Adaboost	The penalty factor *c* ∈ {2^‐5^, 2^‐4^, 2^‐3^, ⋯, 2^3^, 2^4^, 2^5^}, the kernel parameter *δ* ∈ {2^‐5^, 2^‐4^, 2^‐3^, ⋯, 2^3^, 2^4^, 2^5^}
LMPROJ	The regularization parameters *τ* ∈ {0.25, 0.1, ⋯, 200}, *σ* ∈ {0.25, 0.1, ⋯, 200}, the kernel parameter *δ* ∈ {0.25, 0.1, ⋯, 200}
TDLSR	The regularization parameter *λ* ∈ {10^−4^, 10^−3^, 10^−2^, 10^−1^, 10^0^, 10, 10^2^}, *η* ∈ {10^−4^, 10^−3^, 10^−2^, 10^−1^, 10^0^, 10, 10^2^}

**Table 3:** The construction of experimental datasets.

The subdataset	Source domain	Target domain
D1	AE-each 75	AE-each 25
D2	BDE-each 75	BDE-each 25
D3	ABCD-each 75	ABCD-each 25
D4	BCDE-each 75	BCDE-each 25
D5	BE-each 75	BC-each 25
D6	ACE-each 75	BCE-each 25
D7	ADE-each 75	BDE-each 25
D8	ACDE-each 75	BCDE-each 25

**Table 4:** Experimental results based on nontransfer learning algorithms.

Datasets	LSR	SRC	RLR	DLSR	SVM
D1	0.87	0.84	0.89	0.90	0.82
D2	0.79	0.72	0.81	0.80	0.74
D3	0.71	0.62	0.72	0.72	0.68
D4	0.71	0.64	0.70	0.72	0.66
D5	0.82	0.78	0.83	0.84	0.78
D6	0.73	0.60	0.72	0.74	0.61
D7	0.70	0.60	0.70	0.72	0.59
D8	0.63	0.53	0.65	0.64	0.50

**Table 5:** Experimental results based on transfer learning algorithms.

Datasets	Au-SVM	TSVM	Tr-Adaboost	LMPROJ	TDLSR
D1	0.90	0.92	0.89	0.92	0.96
D2	0.82	0.85	0.82	0.84	0.90
D3	0.72	0.75	0.73	0.76	0.83
D4	0.72	0.75	0.71	0.78	0.82
D5	0.85	0.86	0.84	0.87	0.91
D6	0.75	0.78	0.75	0.80	0.83
D7	0.71	0.78	0.74	0.78	0.83
D8	0.68	0.70	0.66	0.70	0.73

**Table 6:** Experimental results based on nontransfer learning algorithms with 15% noise in the source domain.

Datasets	LSR	SRC	RLR	DLSR	SVM
D1	0.83	0.80	0.84	0.85	0.77
D2	0.74	0.67	0.76	0.76	0.70
D3	0.66	0.59	0.68	0.69	0.65
D4	0.66	0.61	0.67	0.68	0.63
D5	0.78	0.74	0.80	0.80	0.74
D6	0.65	0.56	0.69	0.69	0.58
D7	0.64	0.57	0.67	0.71	0.56
D8	0.60	0.50	0.62	0.61	0.47

**Table 7:** Experimental results based on transfer learning algorithms with 15% noise in the source domain.

Datasets	Au-SVM	TSVM	Tr-Adaboost	LMPROJ	TDLSR
D1	0.87	0.86	0.89	0.89	0.93
D2	0.79	0.79	0.82	0.81	0.87
D3	0.69	0.70	0.72	0.73	0.80
D4	0.69	0.68	0.72	0.75	0.79
D5	0.82	0.81	0.83	0.84	0.88
D6	0.72	0.72	0.75	0.77	0.80
D7	0.68	0.71	0.75	0.75	0.80
D8	0.65	0.63	0.67	0.67	0.70

## Data Availability

The labeled dataset used to support the findings of this study are available from the corresponding author upon request.
